# Privacy, Data Sharing, and Data Security Policies of Women’s mHealth Apps: Scoping Review and Content Analysis

**DOI:** 10.2196/33735

**Published:** 2022-05-06

**Authors:** Najd Alfawzan, Markus Christen, Giovanni Spitale, Nikola Biller-Andorno

**Affiliations:** 1 Institute of Biomedical Ethics and History of Medicine University of Zurich Zurich Switzerland

**Keywords:** mHealth, women’s health, ethics, privacy policy, data sharing, privacy, data security, data transparency, femtech, mobile apps, mobile health

## Abstract

**Background:**

Women’s mobile health (mHealth) is a growing phenomenon in the mobile app global market. An increasing number of women worldwide use apps geared to female audiences (female technology). Given the often private and sensitive nature of the data collected by such apps, an ethical assessment from the perspective of data privacy, sharing, and security policies is warranted.

**Objective:**

The purpose of this scoping review and content analysis was to assess the privacy policies, data sharing, and security policies of women’s mHealth apps on the current international market (the App Store on the Apple operating system [iOS] and Google Play on the Android system).

**Methods:**

We reviewed the 23 most popular women’s mHealth apps on the market by focusing on publicly available apps on the App Store and Google Play. The 23 downloaded apps were assessed manually by 2 independent reviewers against a variety of user data privacy, data sharing, and security assessment criteria.

**Results:**

All 23 apps collected personal health-related data. All apps allowed behavioral tracking, and 61% (14/23) of the apps allowed location tracking. Of the 23 apps, only 16 (70%) displayed a privacy policy, 12 (52%) requested consent from users, and 1 (4%) had a pseudoconsent. In addition, 13% (3/23) of the apps collected data before obtaining consent. Most apps (20/23, 87%) shared user data with third parties, and data sharing information could not be obtained for the 13% (3/23) remaining apps. Of the 23 apps, only 13 (57%) provided users with information on data security.

**Conclusions:**

Many of the most popular women’s mHealth apps on the market have poor data privacy, sharing, and security standards. Although regulations exist, such as the European Union General Data Protection Regulation, current practices do not follow them. The failure of the assessed women’s mHealth apps to meet basic data privacy, sharing, and security standards is not ethically or legally acceptable.

## Introduction

### Background

Mobile health (mHealth) is defined by the World Health Organization (WHO) as mobile apps and wearable devices used for health care. Software programs that provide health-related services used by mobile phones and tablets are called mHealth apps [1]. Mobile apps were first introduced by Apple and then by Google Play in 2010. Since then, apps have been frequently used by mobile device users [2]. According to Statista, which reports on data related to the number of apps available on the leading app stores, 3.48 million apps were available on Google Play in the first quarter of 2021, and 2.22 million were available on the Apple App Store. Among the most popular apps are those in the category of health and fitness [3]. The growing number of mobile apps, including mHealth apps, has produced a demand for health services and increased access to health information by mobile app users [1].

Women’s health is a field that focuses on the effect of gender on disease and health and encompasses a range of biological and psychosocial issues [4]. Women’s health is broad and consists of several dimensions: sexual and reproductive health (including pregnancy, sexually transmitted diseases, and menopause), physical health and life expectancy (including nutrition, exercise, and weight management), and mental health. The aforementioned dimensions of women’s health are those that are commonly characterized on the mHealth market [5]. In our study, we explored what is available on the market under the topic of women’s health.

Hundreds of thousands of apps provide services for women on the Apple App Store and Google Play. These apps monitor women’s health and bodily functions, including ovulation, pregnancy, breastfeeding, menstrual cycles, physical activities, mental health, mood levels, stress, and sleep [2]. Millions of people worldwide use women’s health apps [6]. The topics covered by women’s health apps include fitness, lifestyle management, nutrition, diet, reproductive health, medication adherence, and disease management. However, fewer apps are directly related to women’s sexual health and fertility than to diet and exercise [7]. A WHO report recognized that reproductive, maternal, newborn, and children’s health have been a priority for mHealth services in alignment with WHO initiatives, such as the Millennium Development Goals and Every Woman Every Child [1]. In the same report, the WHO recommended the use of women’s mHealth apps in rural areas and low-income countries. Notably, low-cost women’s mHealth apps tend to increase their popularity, especially among rural and low-income countries [8].

In the market, *femtech* (*female technology*; ie, technology geared to female audiences) is an industrial term. Femtech refers to technology related to women’s health, such as software, services, diagnostics, or products [9]. The term *femtech* was coined by the cofounder of Clue, one of the most famous fertility-tracking companies. In her blog, Tin [10] stated that “what female health needs through technology is femtech.” Because half of the global population is female, investment in femtech is growing according to demand [9]. Femtech firms have received significant investment funding. In 2012 alone, they attracted US $57 million; this number increased to US $392 million in 2018 and reached US $2.3 billion in 2020 [11]. This has led to the design of a business model that focuses on individual empowerment involving self-designated women’s health technologies. Women-centered technology is a new concept that has been gaining popularity in the market and has been related to the increased observability of women’s health issues [12]. This huge growth and expansion in the femtech market comes with the price that some of these apps use the data they collect to generate profit. This occurred, for example, in the case of Bounty UK, a pregnancy and parenthood website and app; the UK Information and Commissioner’s Office found that the company supplied and sold data related to pregnant women, new mothers, and infants to a third party “without being fully clear with people that it might do so” [13].

In the sociocultural context, women’s bodies have always been characterized as fluctuating and requiring a high amount of self-regulation. Technology has become a tool for women to oversee their bodies and health [2]. In addition, women are considered to carry the most responsibility in the reproductive health process, from preventing pregnancy to monitoring it until delivery [14]. Motherhood, as in the sociocultural structure, can still affect women in terms of shaming and blaming, including domestic violence, which is strongly associated with unwanted pregnancy and abortion [6]. Furthermore, a lack of knowledge about fertility and the cultural reservation around it encourages women to use mHealth apps to obtain more clarity and awareness in this domain without needing to ask publicly [15]. Sociocultural norms make women more vulnerable in the new tech era [12]. Issues related to women’s bodies that are taboo in some cultures influence the demand for and use of women’s mHealth apps. In most reserved, family-oriented societies, women are expected to conceive a child. Women use these apps as an alternative method to protect themselves from cultural shame [16]. However, stigma about abortion and pregnancy leads some ideological campaigns to use these data to prevent women from obtaining help [6]. Women are under surveillance in some political structures; some states keep track of women’s periods to become aware of any acts of abortion that could be indicated from this information [17]. For example, the Missouri government kept records of women’s periods in clinics to flag any abortion attempts [18]. Moreover, the *ideal* body image of women—an image that is thin but robust, sporty, and sexualized—has been commercialized, influencing the femtech industry to design apps for women that encourage women to strive toward this body image. This has provoked the need and increased the demand for such apps [2]. Women dealing with all these issues are placed in a vulnerable position because they are considered responsible for infertility, are pressured to conceive a child at a certain age or prevent pregnancy, and feel the need to maintain a certain body image [6]. In summary, the need and responsibility of women to conceive a child, prevent pregnancy, or obtain an abortion generates a high demand for women to use these apps. Cultural shame of women’s infertility or weight management leads them to use these apps as a safe zone. Therefore, the following question is raised: Are these apps valid and secure, and are they a safe zone for women? This question was addressed through our study.

In general, personal and health-related data that could be collected in mHealth apps raise ethical concerns, particularly in terms of data privacy, sharing, and security. However, the type of data collected in femtech is typically sensitive, intimate data [17]. Furthermore, women’s mHealth apps are accessible and used on a global level. The practices are set in different cultures and backgrounds [12]. Modern technology has been affected by commercialism and masculinist ideologies [16]. Women’s mHealth apps are mainly commercial, and the data they collect are circulated among different agencies, generating profit for these apps [6]. User consent, especially about sharing data in general or with a third party, is a concern for women’s privacy. Women, as end users of these apps, typically share their personal, health, and intimate data. Research indicates that end users do not have full awareness of what their consent entails [17]. In femtech, the concerns about data privacy and sharing with the commercial agendas of these apps, who accesses the data, and how it is used are complicated and unclear. In addition, in the sociocultural context, women’s vulnerability related to privacy risk by mismanagement and misuse of these data is highly alarming [6]. In this study, we assessed the current practices of the most popular apps in terms of privacy and data sharing.

The concept of women’s health and the case of mHealth is our focus in this report. The spectrum of women’s health encompasses more than just reproductive health and pregnancy. However, most previous studies have focused on reproductive health, pregnancy, and ovulation rather than on women’s health in general. As a result of the increasing number of women using health apps, as well as the increased number of women’s health apps available, we directed our focus to women’s health for this study. As women’s health has become digitalized in the form of femtech mHealth apps, the primary concern has been privacy and data protection [13].

### Privacy, Data Sharing, and Data Security Policies

In total, 3 main concerns arise when considering the ethical implications of mHealth apps: data privacy, data security, and data sharing.

#### Data Privacy

Data privacy is the right of users to control how their information is collected, managed, and used. Data privacy is widely recognized as an essential freedom [19], and respect of data privacy is increasingly regulated at national and international levels, such as by the General Data Protection Regulation (GDPR) in Europe and the Health Insurance Portability and Accountability Act in the United States [20]. Kotz [21] pointed out 25 subcategories of threats to data privacy in mHealth apps, which fall into three main categories: misuse of users’ identities, unauthorized access to data, and unauthorized disclosure of data. A recent scoping review by Nurgalieva et al [22] delineated further criteria for assessing privacy and privacy-related measures, including data ownership, confidentiality, permission systems, auditability, consent, notice of use, disclosure, authenticity, anonymization, data retention, and data access mechanisms.

#### Data Sharing and Data Security

The concepts of privacy and data security partially overlap. Data security is a means to ensure the privacy of users’ data; however, as pointed out by Nurgalieva et al [22], “while security relates to protection against unauthorized access to data, privacy is an individual’s right to maintain control over and be free from intrusion into their private data and communications, and relates to trust in mHealth services.” Data security can thus be defined as the set of procedures and safeguards established to ensure that only authorized users can access a set of data. Assessing data security practices allows for an understanding of how strictly data privacy rights are enforced.

Unauthorized access and data security are not the only issues at stake when considering data privacy. Health-related data gathered from a user can be shared with third parties in various ways. For example, the user may share information with their physician, insurance company, family, and friends, similar to how other information is shared in social networks. This is already happening—many companies offering direct-to-consumer genetic analyses for discovering ancestry or health-related information, such as the presence of genetic markers associated with specific diseases, already offer different degrees of data sharing functions, including the option to share personal data with third parties. Personal health-related data can also be shared in aggregated and anonymized forms for research purposes. This was the case in the Genographic Project [23], a genetic anthropological population study launched in 2005 by the National Geographic Society.

In summary, mHealth apps enable the widespread collection of a wealth of health-related information. Assuming that data privacy and data protection are fundamental human rights, including the right to understand and control which personal data are collected, who collects them, and how and by whom they are used, it is imperative to understand what privacy rights are recognized in practice and how they are enforced.

## Methods

### Overview

The following sections describe the methodology by which women’s mHealth apps were screened, selected, and analyzed regarding their privacy, data sharing, and data security policies. The scoping review followed the methodology introduced by Arksey and O’Malley [24] and was adapted for this app review.

The scoping review protocol was developed by the first author (NF) in cooperation with the second author (MC) in November 2020. The protocol determined the procedure for the initial app search, screening, selection, and analysis. First, the database search and app selection guided by the protocol are described. Second, the screening and selection procedure, which yielded a total of 23 apps that were subject to a refined analysis, is outlined. Third, the analysis schema applied to the selected apps is explained.

### Initial Search for Women’s mHealth Apps

The purpose of this scoping review and content analysis was to evaluate and assess the privacy, data sharing, and data security policies of popular, publicly available women’s mHealth apps on the Apple App Store and Google Play markets, which are considered the largest app markets. As outlined in the *Introduction* section, a considerable number of available apps focused on women’s health and functions (ie, femtech apps).

Therefore, as a first step, appropriate keywords that characterize femtech apps were identified. The keywords were based on our literature search, which described the topic of our scoping review, as explained in the *Introduction* section. Search syntaxes were developed that aligned with a general understanding of women’s mHealth. Different combinations of search terms were tested, starting with a more extensive keyword set to identify a search string that yielded a broad set of results while remaining adequately specific. The primary database search aimed to minimize the number of false-negative results (ie, missing important apps) at the expense of considerable false-positive results (ie, apps that would later be screened out because they did not satisfy the purpose of the analysis). Textbox 1 presents the resulting search string; the 2 components were combined using the OR function.

The search focused on apps available in either the Apple App Store or Google Play. The search procedure made use of the mobile app database 42matters, a private company that provides app intelligence and mobile audience data. In the database, the search strings were categorized to be more specific. In the database search interface, we applied our search terms as detailed in Textbox 1. The database provided more specific filters for searching. The first filter was applied in the search field for *description*, *developer name*, and *title*. For the second filter, the Interactive Advertising Bureau (Interactive Advertising Bureau categories are an industry-standard taxonomy for content categorization that was used by the database), *medical health* was chosen. The third filter was the genre, for which *medical* and *health and fitness* were selected. The fourth filter was the match style of words, for which *exact match* was chosen.

The search performed by the first author (NF) in January 2021 yielded a total of 136 apps from which various pieces of information were collected to allow further screening of the apps (Table 1).

**Search strings used in the database search.**

**Central notion and search string**

- Focus on females
  woman OR women OR feminine OR female
- Focus on health
  health OR medical OR medicine

**Table 1:** Information collected from identified apps.

Information type	Description
App name	Name of the app on the market
Description	Description of the app provided by the developers
Downloads	Download frequency; orders of magnitude: 500, 1000, 5000, 10,000, 50,000, 100,000, 500,000, 1,000,000, 5,000,000, 10,000,000, 50,000,000, and 100,000,000. For screening and selection, the logarithm to the base 10 of the download numbers was used because most download information was available only in orders of magnitude, as explained in the *Methods* (*Screening and Selection* section)
Rating	User rating. Mean rating (between 1 and 5; 1: lowest, 5: highest)
Title	Title of the app
Specific search terms	All keywords that categorize the app
Developer	Developer’s name
Rating count	Number of ratings the app has received from users. For screening and selection, the logarithm to the base 10 of the rating frequency was used
Language (default)	Languages that have been provided by the app
Market status	Whether the app has been published on the market
Website	The app’s website
Interactive Advertising Bureau category	Interactive Advertising Bureau categories are an industry-standard taxonomy for content categorization used by the database

### Screening and Selection

The scoping review focused on apps with certain characteristics such as an adequate number of downloads and a sufficiently large number of ratings by app users. To determine statistically plausible cutoff values for the selection of apps to include in a detailed analysis, a statistical analysis was performed on the connection among download frequency, rating frequency, and actual rating values.

The primary search indicated that the identified apps fell into 2 categories defined by the app providers, Apple App Store and Google Play. *Health and fitness* was the more general category, and *medical* was the more specific category. Of the 163 apps identified in the search, 43 (26.4%) were characterized as *health and fitness* and 93 (57.1%) were characterized as *medical*. The categories did not have sharp boundaries regarding the actual use of the apps; for example, some menstrual cycle tracker apps fell into the *health and fitness* category, whereas others were in the *medical* category. This was because some apps had additional functionalities, making them more health-oriented than others. The analysis relied on the categorization provided by the app providers.

From a statistical point of view, the 2 categories differed substantially concerning download frequency. The mean logarithm of *health and fitness* apps was 4.2 (ie, approximately 15,000 downloads; SD 1.8), whereas the mean logarithm of *medical* apps was 2.9 (ie, approximately 800 downloads; SD 1.5), which presented a significant difference (*P*<.001). The rating frequency distribution displayed a typical long-tail behavior in that many apps yielded only a few ratings and few apps yielded many ratings. Overall, *health and fitness* apps yielded more ratings than *medical* apps; 40% (37/93) of the *medical* apps and 16% (7/43) of the *health and fitness* apps did not produce any ratings. When excluding the apps without ratings and focusing on the mean ratings the apps received, a weak correlation was observed [25] between download quantity and the app ratings (*r*=0.29; *P*=.005) and between the number of ratings and the app ratings (*r*=0.32; *P*=.002). In other words, apps that were downloaded and rated more often had higher ratings. This is crucial, given that apps related to general health were downloaded much more often than medical apps; thus, conducting a direct comparison of both groups regarding quality would not make sense. On the basis of this analysis, we concluded that the 2 categories needed separate cutoff values for choosing the apps for the qualitative analysis.

The main reasons for choosing the cutoff values were that (1) a low download frequency indicated less popular apps, (2) a low rating frequency led to a higher variance in ratings, and (3) download and rating frequencies were strongly correlated. Therefore, rating variances independent of rating frequencies were examined to identify cutoff values separately for the 2 app categories.

For each app, the pair (logarithm of the rating frequency and rating value) was evaluated. These number pairs were ordered in terms of rating frequency (lowest to highest), the rating value variance per bin was calculated for each bin size (starting with 5, ending with 25, sliding window approach), and the distributions were verified visually. For the *health and fitness* apps, a distinct decrease in the rating value variance was observed at bin size 21. This meant that upon reaching the 21st item of the list, the variance dropped. The logarithm of the rating frequency of this bin size was 2.86; thus, the rating frequency should be approximately ≥720. Of the 43 *health and fitness* apps, 16 (37%) fulfilled this criterion. A sensitivity analysis revealed that the apps not chosen in the sequence had 540 and 227 ratings, making it plausible to assume that the result was not strongly affected by a different criterion.

For the *medical* apps, we used the same rating variance value as that used for the *health and fitness* apps. The same reasoning identified bin size 39 as the cutoff value, for which the logarithm of the rating frequency was ≥2, resulting in the selection of 18 apps that had at least 100 ratings. In total, of 163 apps, 34 (20.9%) were selected for further analysis. A sensitivity analysis revealed that the apps had 82, 78, and 45 ratings, supporting the plausibility of the cutoff criterion.

In summary, 34 apps were chosen on the basis of the statistical criterion and were further analyzed using the exclusion criteria in Table 2.

**Figure 1:**
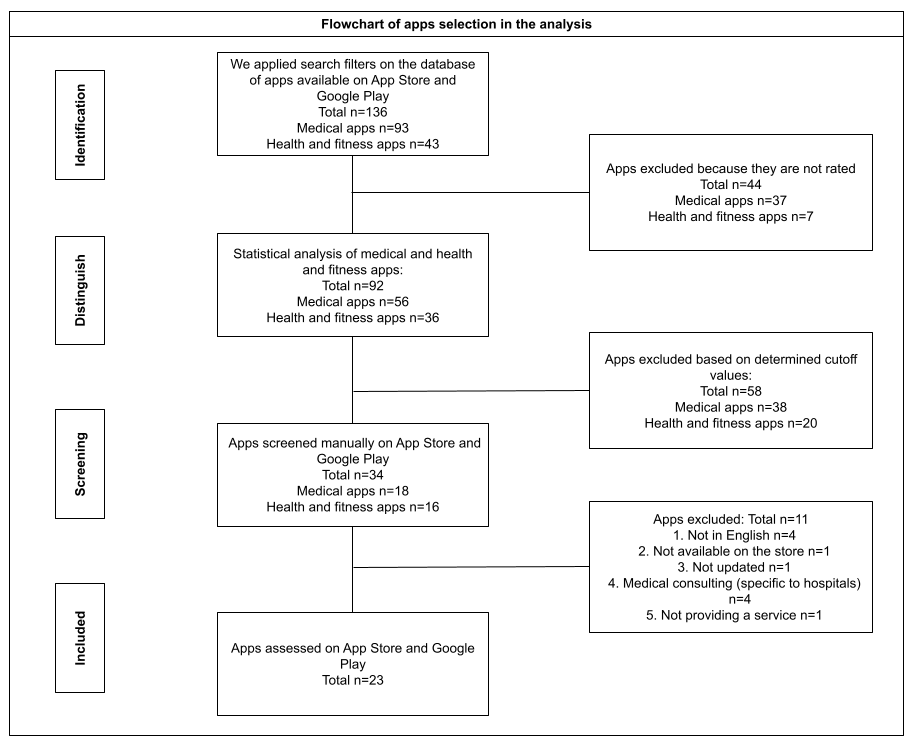
PRISMA (Preferred Reporting Items for Systematic Reviews and Meta-Analyses) flowchart outlining the search and selection procedure.

Using these criteria, 4 medical consulting apps were excluded because users were required to be associated with a specific hospital in a certain region or country. In addition, 4 other apps were excluded because they were not available in English, although the store page showed that they were available in English. Then, 1 app was not available in the Apple App Store at the time of our analysis because there was no update for the iPhone 11 (software version 14.4; Apple Inc). The same app downloaded from Google Play crashed after opening. In addition, 1 app did not provide a service related to women’s health. Lastly, 1 app was no longer available in the store. In summary, of the 34 apps, 11 (32%) were excluded, and 23 (68%) remained for the final analysis. Figure 1 provides an overview of the search and selection process including the number of apps identified.

**Table 2:** Exclusion criteria.

	Description
Language	The app must be available in English.
Time Frame	The app must be functional in the search period (January to March 2021).
Service	The app must provide a service related to women’s health, not only access to information (such as articles or magazines) or a game related to one of the topics described by the search string.

### App Analysis

In the last step, the 23 identified apps were downloaded and assessed independently by 2 reviewers (the first [NF] and third author [GS]). Apps on Google Play were assessed in LDPlayer 4 (Xuanxi International Co), a PC framework software that allows Android apps to run on a computer. LDPlayer 4 emulates a Samsung A908N tablet and uses Android 7.1.2 (security patch October 5, 2017, kernel 3.18.48). Apple App Store apps were analyzed on an iPhone 11 (software version 14.4). All apps were downloaded and tested between February and March 2021.

One of the main differences between the Apple App Store and Google Play is that the Apple App Store has the option to review an app’s privacy policy before downloading the app. This option is not available in Google Play. However, Google Play includes a Pan European Game Information (PEGI) score, which is a rating system developed and intended to assess the appropriateness of video games, considering the presence of bad language, discrimination, drugs, fear, gambling, sex, violence, and in-game purchases [26]. Nevertheless, the PEGI score does not always provide useful information on the appropriateness of apps for certain age groups. PEGI scores can be inconsistent; for example, apps for lung cancer screening and abortion are both rated as PEGI 3. In addition, the PEGI age limit often contradicts age limits specified within privacy policies or terms and conditions.

The downloaded apps were assessed manually by 2 independent reviewers against a variety of user data privacy, data sharing, and security assessment criteria. These criteria, presented in Table 3, were selected from 2 studies that focused on app security and privacy assessments [27,28]. The selected assessment criteria were developed in compliance with the European Union (EU) GDPR. The assessment questions were categorized into several domains: privacy policy, data gathering, data sharing, security, and transparency. Our assessment was based on *yes* and *no* answers. In some cases, it was not clear if the criteria applied, so *not applicable* was used as a response for vague statements or if the question was not answered. In addition, a qualitative portion of the assessment was included for each question, which allowed the reviewers to add comments detailing their observations. These comments are not included in Table 3 but are included in the *Results* section. This review assessed each app’s privacy policy, if it existed, by screening each app manually after downloading. In this analysis step, the apps were evaluated using the assessment questions listed in Table 3.

**Table 3:** Data privacy, sharing, and security assessment results (N=23).

				Value, n (%)
**Privacy policy**				
	**Is the privacy policy available within the app?**			
		Yes	16 (70)	
		No	7 (30)	
	**Is the privacy policy available before downloading the app?**			
		Yes	19 (83)	
		No	4 (17)	
	**Is there a short-form notice (in plain English) highlighting key data practices that are disclosed in detail in the full privacy policy?**			
		Yes	0 (0)	
		No	23 (100)	
	**Is the privacy policy available in any other languages?**			
		Yes	3 (13)	
		No	20 (87)	
	**Are there specifications of the privacy policy for users in certain regions or countries?**			
		Yes	11 (48)	
		No	12 (52)	
	**Is contact information provided for the users’ questions regarding the privacy policy?**			
		Yes	19 (832.6)	
		No	4 (17)	
	**Does the app request explicit consent to start storing** **all user health and sensitive data when an account is created?**			
		Yes	12 (52)	
		No	11 (48)	
**Data gathering**				
	**Is there an age restriction for data collection and account creation for adult services?**			
		Yes	21 (91)	
		No	2 (9)	
	**Does required sensitive data include personal data that directly identifies the person (eg, first name, surname, email, date of birth, and mobile phone number)?**			
		Yes	21 (91)	
		No	2 (9)	
	**Does required sensitive data include health-related personal information?**			
		Yes	23 (100)	
		No	0 (0)	
	**Is an account required to use the app (ie, does the app require a login and password)?**			
		Yes	14 (61)	
		No	9 (39)	
	**Are data collected when a user registers through a web-based account?**			
		Yes	15 (65)	
		No	8 (35)	
	**Are data collected when the app is used?**			
		Yes	11 (48)	
		No	12 (52)	
**Data sharing**				
	**Can the user opt out or withdraw by deleting the app?**			
		Yes	17 (74)	
		No	3 (13)	
		N/A^a^	3 (13)	
	**Can the user delete past data by request?**			
		Yes	14 (61)	
		No	7 (30)	
		N/A	2 (9)	
	**Does the app allow behavior tracking?**			
		Yes	23 (100)	
		No	0 (0)	
		N/A	0 (0)	
	**Does the app allow location tracking?**			
		Yes	14 (61)	
		No	7 (30)	
		N/A	2 (9)	
	**Does the app share users’ data with a third party?**			
		Yes	20 (87)	
		No	0 (0)	
		N/A	3 (13)	
	**Can the user change the sharing settings?**			
		Yes	12 (52)	
		No	9 (39)	
		N/A	2 (9)	
	**Does the app share personal data for research purposes with a third party?**			
		Yes	18 (78)	
		No	4 (17)	
		N/A	2 (9)	
	**Does the app share data with third parties for tracking and analysis?**			
		Yes	15 (65)	
		No	4 (17)	
		N/A	4 (17)	
	**Are personal data shared if required by law?**			
		Yes	20 (87)	
		No	1 (4)	
		N/A	3 (13)	
**Data security and transparency**				
	**Does the app explain how the users’ data security is ensured (eg, encryption, authentication, or firewall system)?**			
		Yes	13 (57)	
		No	8 (35)	
		N/A	2 (9)	
	**Is the app transparent about how it processes data?**			
		Yes	16 (70)	
		No	6 (26)	
		N/A	1 (4)	

^a^N/A: not applicable

## Results

### Overview

Our assessment included 23 women’s mHealth apps. Among the 23 women’s mHealth apps that we analyzed, 16 (70%) were related to fertility health, ovulation or menstrual cycle tracking, and pregnancy; 1 (4%) was related to abortion; 2 (9%) were related to breast and lung cancers; 1 (4%) was related to women’s mental health and self-care; and 3 (13%) were related to women’s health exercises (eg, pelvic floor exercises and weight tracking; Table 4). These categories matched those defined as the dimensions of women’s health in the Introduction section

**Figure 2:**
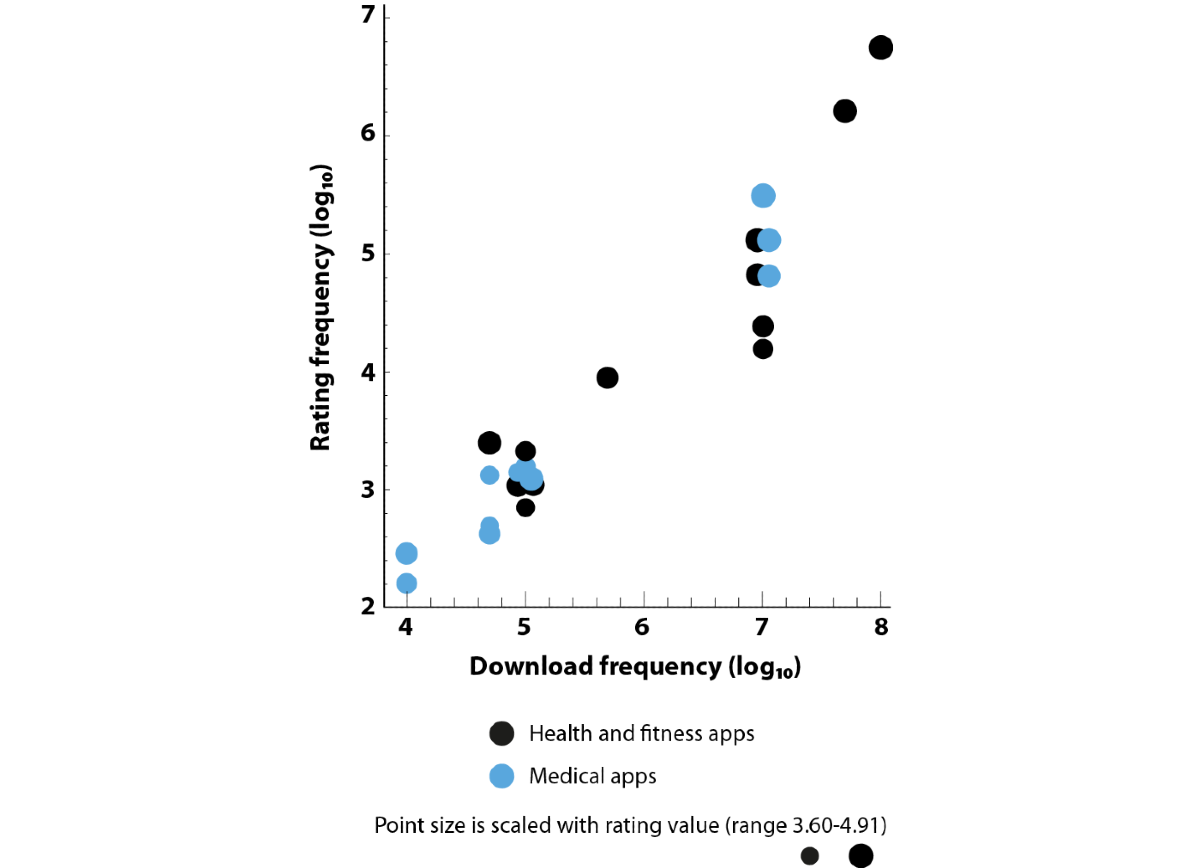
Scatterplot demonstrating the statistical relationship between download frequency and rating frequency of the 23 analyzed apps.

Figure 2 displays the general characteristics of the 23 apps analyzed in this study. We plotted download frequency against rating frequency (log_10_ scale) of *health and fitness* apps (black) and *medical* apps (blue); point size is scaled with the rating value of each app. The figure demonstrates the (expected) strong correlation between download and rating frequency and reproduces the initial finding that *health and fitness* apps are generally more popular than *medical* apps (see the *Screening and Selection* section). The results of the evaluation of data privacy, data sharing, and security assessment are summarized in Table 3.

**Table 4:** Women’s health app taxonomy (N=23).

Category	Apps, n (%)
Fertility health, ovulation or menstrual cycle, and pregnancy	16 (70)
Abortion	1 (4)
Breast cancer and lung screen	2 (9)
Women mental health (self-care)	1 (4)
Exercise (eg, pelvic floor exercises and weight tracking)	3 (13)

### Privacy Policy

In 4% (1/23) of the apps, we found that the privacy policy was available on the store page but not inside the app itself; we assessed the app using only the information available in the Apple App Store. Of the 23 apps, 1 (4%) was available in English only on Google Play but not on the Apple App Store. Therefore, we analyzed the app only on Google Play.

Of the 23 apps reviewed, 7 (30%) did not have a privacy policy available within the app, whereas 4 (17%) had a privacy policy available on the Apple App Store page before downloading the app. As Google Play does not require the privacy policy to be included on the page displayed before downloading the app, Google Play users cannot read the privacy policy beforehand. The other 13% (3/23) of the apps did not have privacy policies either within the app or before downloading the app. In addition, of the 23 apps, 1 (4%) had a privacy policy after creating an account, but the privacy policy was not accessible anywhere else in the app. In 4% (1/23) of the apps, the link led to the privacy policy on the app website. However, on the website, the privacy policy was available on another page. Thus, reaching the privacy policy requires a long process; the user must go through the main website and search for it, and at least four clicks were required to find it. Therefore, users who want to read the privacy policy cannot reach it directly from the app page on the store or in the app itself.

Of the 23 apps analyzed, 11 (48%) provided their services in more than one language, including English, yet their privacy policies were only available in English. Only 13% (3/23) of the apps provided their privacy policies in languages other than English. None of the apps reviewed had a short-form notice (in plain English) highlighting key data practices that were disclosed in detail in the full privacy policy. Only 9% (2/23) of the apps provided options for viewing the privacy policy (summary view or full view) but not in a short-form notice. In addition, of the 23 apps, 1 (4%) included the privacy policy with illustrated pictures, but the privacy policies of the remaining apps were in plain text.

Of the 23 apps, 11 (48%) had specifications in their privacy policies related to certain laws and regulations, such as the California Consumer Privacy Act, the EU GDPR, and the UK Data Protection Act 2018). Of the 23 apps, 4 (17%) did not provide any contact information to address users’ questions regarding the privacy policy, whereas 3 (13%) did not have a privacy policy at all.

A total of 48% (11/23) of the apps did not require explicit consent to the privacy policy. The welcome page of 4% (1/23) of the apps provided an option to read the privacy policy; however, clicking or consenting was not required before entering the app. Among the 52% (12/23) of the apps that required consent, only 8% (1/12) displayed the consent requirement at the welcome page with transparent options (the welcome page provided 4 options for consent, and they had to be accepted to enter and use the app). Another app prompted the user to accept the privacy policy; however, the privacy policy did not exist—it was not available in the app, store, or website. We considered this to be a pseudoconsent. The welcome pages of 3 other apps asked personal and health questions and would not allow the user to move to the next page without filling in all fields, but consent was not required until the second page. One app’s privacy policy had an option to “expressly agree,” but the form was accessible only after registration, whereas another app’s welcome page had a large button to “get started,” under which was written in smaller font that by tapping “get started,” the user was stating “I consent to the privacy policy.”

### Data Gathering

We found that 17% (4/23) of the apps provided different age restrictions on the Apple App Store compared with those on Google Play; of the 23 apps, 3 (13%) showed different ages on the app store pages and in their privacy policies, and 2 (9%) did not provide any age restrictions. Only 35% (8/23) of the apps had privacy policies that stated that users under a certain age should have parental consent.

All apps in the study required the entry of sensitive health-related data and personal information. Of the 23 apps, 1 (4%) also asked for information about the children, such as the child’s age and overall health-related questions. Only 9% (2/23) of the apps did not collect sensitive data, such as personal data that directly identified the user (eg, name, surname, email address, date of birth, and mobile phone number). Of the 23 apps, 11 (48%) collected personal data once the user started using the app without any registration, whereas 9 (39%) required no consent before using the app.

### Data Sharing, Security, and Transparency

Of the 23 apps, 7 (30%) did not provide the user an option to delete past data by request, such as by sending an email, and 3 (13%) did not allow the user to opt out or withdraw by deleting the app. Of the 23 apps, 3 (13%) others did not provide any information on requesting to opt out or withdraw by deleting the app. All apps allowed behavioral tracking, whereas only 4% (1/23) of the apps gave the user the ability to opt out. Of the 23 apps, 7 (30%) did not allow location tracking, and 2 (9%) other apps did not provide any information about location tracking. Of the 23 apps, 20 (87%) shared user data with third parties, 9 (39%) did not require explicit consent to share user data with third parties, and 3 (13%) did not provide any information in their privacy policies and did not require consent related to the sharing of user data with third parties. We found that 78% (18/23) of the apps shared personal data for research purposes, 33% (6/18) of which did not require user consent, and 65% (15/23) of the apps shared data with a third party for tracking and analysis.

In total, of the 23 apps, 20 (87%) shared user information if required by law. Of these 20 apps, 9 (45%) did not require user consent; 2 (10%) did not require user consent and did not provide a clear statement in their privacy policies disclosing whether they share user information; and 1 (5%) did not have a privacy policy. Among the 23 apps reviewed, 16 (70%) were transparent about how they processed the data, and 6 (26%) did not share any information regarding how the data were processed. Of the 23 apps, 8 (35%) did not provide information regarding how users’ data would be secured.

## Discussion

### Principal Findings

The goal of this review and content analysis was to assess the privacy, data sharing, and security policies of women’s mHealth apps on the market. This scoping review was important because of the growing presence and use of such apps in the women’s health domain, as identified by both health sciences and the mHealth app market (ie, femtech).

This review revealed important shortcomings associated with privacy policies and consent practices, especially in the case of women’s mHealth apps. The apps that we analyzed were the most frequently downloaded from the market and had the highest ratings. Through our review and analysis, we found that women’s mHealth apps collected and tracked personal and health data. However, their standard practices did not follow regulations, such as the EU GDPR. Data privacy and protection have been suggested as fundamental human rights. In this review and analysis, we sought to understand the practices of select women’s mHealth apps. Our results revealed poor data privacy protection practices. It is ethically unacceptable that, despite the existence of regulations such as EU GDPR, there are still gaps in data privacy and security practices.

All apps included in our analysis collected personal and health data; however, the option for the user to give consent and read the privacy policy was not always available. The involvement of end users is essential, especially when personal and health data are collected. Not requiring the consent of the end user when collecting sensitive information is an ethical violation. Moreover, the use of a range of women’s mHealth apps is increasing worldwide [7]. Many available apps provide services in multiple languages, which allows them to be used by people who cannot speak English. However, we found that most apps provided their privacy policies only in English. Users who cannot read English are unable to review and understand these privacy policies. Therefore, users may give their consent without reading or understanding the privacy policies of these apps. The right of the end user to access and understand what they provide consent for is a basic right that must be upheld.

The type of data collected by women’s mHealth apps is considered sensitive in general. In some cultures, women’s bodies and health are taboo subjects. Therefore, the collection of women’s personal and health data could have negative consequences in certain areas of the world [12]. Given the sensitive nature of women’s health, women’s mHealth apps should practice increased privacy rather than the poor practices uncovered in this study. Moreover, some women’s mHealth apps collected not only women’s sensitive data but also information on children and infants. These observations demonstrate the complexity of the standard practices of data privacy and consent. Finally, also the age of the users is a factor to consider, as younger women are—because of a generally higher affinity of younger people to health apps—likely to be a big audience for these apps [2]. Adolescence and early adulthood are important phases in the human life span, and the experience of potential violation of privacy on sensitive data can have a considerable impact. Our study was not designed to consider those aspects, but future studies should include the role of age and culture on femtech use.

### Recommendations

It is evident that poor data privacy practices do not deter users, as demonstrated by the high number of users of apps with unsatisfactory privacy policies. This generates the following 2 questions. First, are women as end users aware of the privacy practices of the mHealth apps to which they provide their personal and health data? Second, do they know how their data will be used? Future studies should focus on measuring women’s awareness of mHealth apps’ data privacy and sharing practices. It is critical to understand what data women share with mHealth apps, whether they understand the apps’ privacy policies in their current forms and whether alternative forms of the apps’ privacy policies should be made available.

Consumers are typically unable to assess privacy, data sharing, and data security policies. More stringent regulations would require apps to adhere to defined standards for their policy descriptions and how they may or may not prompt users to accept their policies. Although not an ideal solution, privacy checkups should be easily accessible so that users can better understand policies in the absence of stricter regulations. Despite current regulations, such as the GDPR, protocols should be improved to enable users to examine and understand policies. An educational study on the relevance of data protection, particularly with artificial intelligence, was conducted on pooled personal data. Further studies could be conducted for cases in which clear and transparent privacy policies do not exist. We recommended surveying women with a short-form privacy policy to illustrate the main points while providing access to the full form. Privacy policies should be improved to include illustrated figures and photos in a shorter form to aid in the end user’s awareness and understanding. This is imperative for understanding the future design of women mHealth apps.

The Apple App Store and Google Play, which are considered the largest app providers, should require that apps follow the regulations. It was observed that the Apple App Store requires privacy policies to be displayed on the apps’ store pages; however, this is not the case for Google Play. The Apple App Store and Google Play should be responsible for such regulations, rather than only reaping the benefits associated with mHealth apps. For instance, an app that provides services in multiple languages should be required to also provide its privacy policy in those languages.

### Conclusions

This review and content analysis examined the most popular women’s mHealth apps on the market. The market for women’s mHealth apps is large, with millions of users worldwide; the mHealth app industry is growing, and the number of available apps is increasing. Women’s health is a complicated topic in many ways. In our analysis, we found that the most popular women’s mHealth apps on the market have poor data privacy, sharing, and security practices. Although regulations exist, such as the EU GDPR, current practices do not follow them. Moreover, other studies conducted on various dimensions of women’s mHealth apps, such as on reproductive health, pregnancy, and ovulation, have concluded that those apps have poor practices in terms of privacy, data sharing, and data security [6,17]. These poor data privacy and sharing practices generate concern regarding health and personal data. The studied mHealth apps lack basic data privacy and security practices, which is unacceptable, both ethically and legally.

## Abbreviations

EU: European Union
femtech: female technology
GDPR: General Data Protection Regulation
mHealth: mobile health
WHO: World Health Organization
